# Whole-Body Vibration Partially Reverses Aging-Induced Increases in Visceral Adiposity and Hepatic Lipid Storage in Mice

**DOI:** 10.1371/journal.pone.0149419

**Published:** 2016-02-17

**Authors:** Aaffien C. Reijne, Jolita Ciapaite, Theo H. van Dijk, Rick Havinga, Eddy A. van der Zee, Albert K. Groen, Dirk-Jan Reijngoud, Barbara M. Bakker, Gertjan van Dijk

**Affiliations:** 1 Groningen Institute for Evolutionary Life Sciences, Neurobiology, Unit of Behavioral Neurosciences, University of Groningen, Nijenborgh 7, NL-9747AG Groningen, The Netherlands; 2 Department of Pediatrics, Center for Liver, Digestive and Metabolic Diseases, University of Groningen, University Medical Center Groningen, Hanzeplein 1, NL-9713GZ Groningen, The Netherlands; 3 Systems Biology Centre for Energy Metabolism and Ageing, University Medical Center Groningen, University of Groningen, Antonius Deusinglaan 1, NL-9713AV Groningen, The Netherlands; 4 Department of Laboratory Medicine, University of Groningen, University Medical Center Groningen, Hanzeplein 1, NL-9700RB Groningen, The Netherlands; 5 Groningen Institute for Evolutionary Life Sciences, Neurobiology, Unit of Molecular Neurobiology, University of Groningen, Nijenborgh 7, NL-9747AG Groningen, The Netherlands; 6 ESRIG Center for Isotope Research, University of Groningen, Nijenborgh 4, NL-9747AG Groningen, The Netherlands; West Virginia University School of Medicine, UNITED STATES

## Abstract

At old age, humans generally have declining muscle mass and increased fat deposition, which can increase the risk of developing cardiometabolic diseases. While regular physical activity postpones these age-related derangements, this is not always possible in the elderly because of disabilities or risk of injury. Whole-body vibration (WBV) training may be considered as an alternative to physical activity particularly in the frail population. To explore this possibility, we characterized whole-body and organ-specific metabolic processes in 6-month and 25-month old mice, over a period of 14 weeks of WBV versus sham training. WBV training tended to increase blood glucose turnover rates and stimulated hepatic glycogen utilization during fasting irrespective of age. WBV was effective in reducing white fat mass and hepatic triglyceride content only in old but not in young mice and these reductions were related to upregulation of hepatic mitochondrial uncoupling of metabolism (assessed by high-resolution respirometry) and increased expression of uncoupling protein 2. Because these changes occurred independent of changes in food intake and whole-body metabolic rate (assessed by indirect calorimetry), the liver-specific effects of WBV may be a primary mechanism to improve metabolic health during aging, rather than that it is a consequence of alterations in energy balance.

## Introduction

Declining mortality due to advances in the health care system results in a right-shift in the age distribution of humans in industrialized societies. The global share of older people (aged 60 years or over) increased from 9.2% in 1990 to 11.7% in 2013 and will continue to grow as a proportion of the world population, reaching 21.1% in 2050 [1]. This is expected to have major social and economic consequences, because the population as a whole may not necessarily be healthier than before.

Aging is characterized by a gradual decline in muscle mass with a concomitant increase in fat mass and abdominal circumference (i.e. obesity) [2], and could be a consequence as well as an underlying cause of the disintegrative metabolism seen with aging. The progressive loss of muscle mass and strength (sarcopenia) increases the risk of disability and poor quality of life [3], and increased fat mass constitutes an important risk factor for the development of type 2 diabetes and cardiovascular disease [2]. Maintenance of regular physical activity has been shown to attenuate the age-related decline in muscle mass and strength [4] as well as fattening of the body [5], and to preserve metabolic functioning [6], this way contributing to improved overall health in older adults. However, many traditional forms of physical activity may not be suitable for elderly due to debilitation, increased risk of injury, or lack of motivation.

Over the past decade, whole-body vibration (WBV) has become an increasingly popular training method in humans for enhancement of athletic performance [7], but also for therapeutic purposes in the field of geriatric rehabilitation [8]. In WBV training, vibration stimuli are delivered to the body via the vibration platform or chair. Mechanical vibrations have been shown to induce reflex muscle contractions, which result from activation of sensory receptors in the muscle (muscle spindles) leading to the activation of muscle motor units [9,10]. Moreover, vibration stimuli may influence systemic hormone levels and tissue perfusion due to direct mechanical stimulation or as the result of altered neuromuscular activity [11]. The acute WBV effects have been shown to include increased muscle activity, blood flow and muscle and skin temperature [12]. The data on the hormonal responses are contradictory, showing either an increase in the testosterone and growth hormone levels [13] or no change [14], with a consistent finding of lower cortisol levels immediately after WBV [13,14]. WBV can also be safely applied chronically, with daily sessions over several weeks or months. Chronic WBV can be seen as a passive exercise modality. Similarly to regular exercise, there is a direct increase in energy expenditure arising from the exposure to vibration [15], which may underlie body weight loss as shown in several studies [16–19], although others claim that an increase in the energy demand in response to WBV alone may not be sufficient to reduce body fat mass [20]. The combination of WBV with other treatments, like diet [16] or endurance training [21] clearly improves the individual’s health status. Age appears to be an important determinant for the outcome of WBV treatment, with more beneficial effects observed in older adults compared to young healthy individuals. For example, a study in untrained young women receiving WBV showed no changes in body composition [22], while 24 weeks of WBV training in postmenopausal women caused a significant decrease in body fat mass [17]. Similarly, 8 weeks of WBV in young Wistar rats actually increased body weight in the vibration versus the control group [23], while in old rats 12 weeks of WBV resulted in a reduction of body fat mass and lowering of leptin levels compared to controls [24].

Very little is known about how WBV affects whole-body energy balance characteristics, and it is certainly not clear which mechanisms underlie the age-dependent responses to WBV. In the present study we therefore characterized energy balance responses to WBV treatment in young and old mice and investigated several aspects of fuel metabolism at the whole-body and organ level. We observed that WBV treatment had a stronger effect on lipid than on glucose metabolism. WBV was effective in reducing white fat mass and hepatic triglyceride content in old but not in young mice and these reductions were related to upregulation of mitochondrial uncoupling protein 2 (UCP2) and increased mitochondrial uncoupling assessed by high-resolution respirometry.

## Materials and Methods

### Animals and experimental protocol

Male C57BL6/JOlaHsd mice (n = 40) (Harlan Netherlands BV, Horst, The Netherlands) were housed singly on a 12hr:12hr light:dark cycle in a temperature-controlled environment (22±1°C) with *ad libitum* access to standard lab chow (RMH-B 2181, HopeFarms BV, Woerden, NL) and water. Mice were tested in two age categories (n = 20/group), namely at young age (at start of treatment 2.3 ± 0.0 months) and at old age (at start of treatment 21.6 ± 3.1 months). These groups were further randomly subdivided into an experimental group that received WBV (n = 10), and into a control group that was exposed to sham-training (n = 10). The vibration protocol lasted for 14 weeks during which mice in the experimental groups were placed individually in boxes on a custom-made vibrating plate (total 27x42 cm, each individual box 6.3x7.3 cm) for ten minutes a day, five days a week. The vibration frequency was 30Hz [25] with an amplitude of 1.9*g* [26,27]. The control group was placed on the same vibrating plate for the same time period, but the apparatus was not switched on. Body weight and food intake were assessed three times a week. All methods were approved by, and are in agreement with the regulations of the Institutional Animal Use and Care Committee of the University of Groningen. These regulations are consistent with the guidelines for the care and use of laboratory animals as described by the U.S. National Institutes of Health.

### Indirect calorimetry

During the ninth week of WBV training mice were placed within their home cage in a respirometry chamber where oxygen consumption (VO_2_, ml/h) and carbon dioxide production (VCO_2_, ml/h) were recorded for each individual mouse for 24 hours. The eight-channel open circuit indirect calorimetry system has been described earlier by Oklejewicz and colleagues [28]. The respiratory quotient (RQ) was calculated as VCO_2_/VO_2_. Energy expenditure (EE, kJ/h) was calculated according to the equation of Ferrannini [29].

$$\text{Energy expenditure}\;\left(\text{kJ}/\text{hr}\right)=\left(\left(\text{RQ -}\;0.7\right)/0.3\;*\;473\right)+\left(\left(1.0\;\text{- RQ}\right)/0.3\;*\;439\right)\;*\;{\text{VO}}_{2}\left(\text{mol}/\text{hr}\right)$$

Where oxidation (by one mol O_2_) of carbohydrates and fats yields respectively 473 and 439 kJ. Carbohydrate and fat oxidation rates (g/h) were calculated according to the equations of Lusk [30], protein oxidation was not calculated because urine was not collected during this measurement.

$$\begin{array}{l} \text{Carbohydrate oxidation}\;\left(\text{g}/\text{hr}\right)=\left(\left(94.017\;*\;{\text{VCO}}_{2}\left(\text{mol}/\text{hr}\right)\right)\;\text{-}\;\left(66.239\;*\;{\text{VO}}_{2}\left(\text{mol}/\text{hr}\right)\right)\right)\;*\;1000\\ \text{Lipid oxidation}\;\left(\text{g}/\text{hr}\right)=38461\;*\;\left({\text{VO}}_{2}\left(\text{mol}/\text{hr}\right)–{\text{VCO}}_{2}\left(\text{mol}/\text{hr}\right)\right) \end{array}$$

These formulas are derived from the notion that 0.036 mol and 0.088 mol of O_2_ are necessary to oxidize unit masses of carbohydrates and fats, respectively, and 0.036 mol and 0.062 mol of CO_2_ are produced upon oxidation of unit masses of carbohydrates and fats.

### Hepatic carbohydrate flux measurements *in vivo*

After 11–12 weeks of WBV, mice were anesthetized using a 2% isoflurane inhalation mixture and received a catheter, which was placed into the jugular vein and exteriorized on the skull. Post-operative injection of buprenorphine was used as analgesic. Then mice were allowed to recover for one week, during which they did not receive WBV. *In vivo* hepatic carbohydrate fluxes were determined in conscious mice as described previously [31]. In brief, mice were fasted for nine hours and then a blood sample was collected for the determination of fasting blood glucose and plasma insulin concentrations via retro-orbital bleeding under short anesthesia. After attaining consciousness mice were infused with a solution containing [U-^13^C]-glucose (1.25 mg/ml), [1-^2^H]-galactose (3 mg/ml), [U-^13^C] glycerol (7.5 mg/ml) (Cambridge Isotope Laboratories, Andover, MA, USA), and paracetamol (1.0 mg/ml) (Sigma-Aldrich, Zwijndrecht, The Netherlands) at a rate of 0.6 ml/h for six hours. At hourly intervals, the blood spots and urine samples were collected on filter paper for GC-MS analysis. Blood glucose levels were measured in blood samples obtained via tail-tip bleeding using a handheld Lifescan OneTouch UltraEasy glucose meter (LifeScan Inc., Milpitas, USA). The filter paper with blood spots and urine samples were air-dried and stored at room temperature until further analysis. Hepatic carbohydrate fluxes were calculated using mass-isotopomer distribution analysis (MIDA) as previously described [31,32]. After this experiment, mice quickly recovered lost body weight and WBV treatment continued.

### Blood and tissue collection and determination of body composition

At the end of the experimental protocol (14 weeks since the beginning of WBV treatment), mice were again anesthetized using a 2% isoflurane inhalation mixture and blood was collected through cardiac puncture and transferred into EDTA-containing tubes. Mice were euthanized by cervical dislocation. Liver and both quadriceps muscles were quickly excised and half of the liver and one quadriceps muscle were snap-frozen in liquid nitrogen and stored at -80°C until further biochemical analyses. The other half of the liver and the second quadriceps muscle were kept fresh and used for the isolation of mitochondria. The remaining organs were excised, weighed and stored at -80°C until determination of body composition. Dry and dry lean organ masses were determined by drying the organs to a constant mass for 14 days at 60°C followed by fat extraction with petroleum ether (Boom BC, Meppel, The Netherlands) in a custom-made soxhlet apparatus. Blood was centrifuged at 4000 g for 10 min at 4°C, the resulting plasma was divided in aliquots, and stored at -80°C until further analyses.

### Isolation of mitochondria and high-resolution respirometry (HRR)

Mitochondria were isolated from liver and skeletal muscle (quadriceps) by differential centrifugation procedure as described previously [33,34]. Protein content was determined using a BCA protein assay kit (Pierce, Thermo Fisher Scientific Inc., Rockford, IL, USA).

The O_2_ fluxes in isolated liver and skeletal muscle mitochondria were measured at 37°C in a two-channel high-resolution Oroboros oxygraph-2 k (Oroboros, Innsbruck, Austria). The assay medium (MiR05) contained 110 mM sucrose, 60 mM potassium lactobionate, 20 mM taurine, 20 mM HEPES, 0.5 mM EGTA, 10 mM KH_2_PO_4_, 3 mM MgCl_2_, and 1 mg/ml bovine serum albumin, at pH 7.1. The oxidizable substrates were: (i) 5 mM pyruvate plus 2 mM malate, or (ii) 25 μM palmitoyl-CoA plus 2 mM L-carnitine plus 2 mM malate. The maximal ADP-stimulated O_2_ flux (state 3) was achieved by adding 1.5 U/ml hexokinase, 12.5 mM glucose and 1 mM ATP. The basal O_2_ flux (state 4) was determined after blocking ADP phosphorylation with 1.25 μM carboxyatractyloside. Data acquisition and analysis were performed with DatLab software version 4.2 (Oroboros, Innsbruck, Austria).

### Tissue and plasma analyses

Pieces of frozen liver and quadriceps muscles were weighed. For triglyceride determination 10% homogenates (w/v) were prepared in ice-cold PBS (pH 7.4). Lipids were extracted according to Bligh and Dyer [35]. Triglyceride content was determined with a commercial kit (Roche Diagnostics, Mannheim, Germany) according to manufacturer’s recommendations. Hepatic glycogen content was determined according to Lavoinne and colleagues [36]. Plasma leptin concentrations were determined with commercial kits (Millipore). Plasma insulin concentrations were determined with a commercial radioimmunoassay (RIA) kit (Linco Research). Plasma cholesterol and triglyceride (TG) concentrations were determined with a Cholesterol kit (Roche Diagnostics) and Triglyceride/Glycerol blanked kit (Roche Diagnostics), respectively. Plasma free fatty acid (FFA) concentrations were determined using a NEFA C kit (Wako Chemicals, Neuss, Germany).

### Mitochondrial DNA (mtDNA) copy number

Genomic DNA was isolated from ~20 mg of tissue using GenElute Mammalian Genomic DNA Miniprep Kit (Sigma-Aldrich, Zwijndrecht, The Netherlands). Relative mtDNA copy number was determined by assessing the copy number of the mitochondrial-genome-encoded 16S ribosomal RNA gene (*mt-Rnr2*) relative to a single-copy nuclear glyceraldehyde-3-phosphate dehydrogenase gene (*Gapdh*) by real-time PCR. Real-time PCR was performed in MicroAmp optical 96-well plates in the StepOne Real-Time PCR system (Applied Biosystems). The reaction volume of 15 μl contained 10 ng of genomic DNA, forward and reverse primers (0.25 μM each) and 1× SensiMix SYBR Hi-ROX mastermix (cat. no. QT605-05, Bioline). Primers were designed using Primer Express software version 3.0 (Applied Biosystems). Primer sequences were: *mt-Rnr2* forward – 5’-TTAACCCAACACCGGAATGC-3’, *mt-Rnr2* reverse – 5’-GGGTTCTTGTTTGCCGAGTTC-3’, *Gapdh* forward – 5’-TTTGTTGTGGTACGTGCATAGCT-3’, *Gapdh* reverse – 5’-GCTATCTCATGTTCTTCAGAGTGGAA-3’. Relative mtDNA copy number was calculated with the ΔΔCt method.

### Citrate synthase activity

Tissues were homogenized in ice cold PBS (pH 7.4). Homogenates were sonicated for 30 s in the pulse mode (pulse duration 1 s, interval between the pulses 1 s, power input 10 W) on ice, followed by 10 min centrifugation at 1000 g, 4°C. Citrate synthase activity in the supernatant was determined spectrophotometrically according to Srere and colleagues [37].

### Immunoblotting

Equal amounts (10 μg) of mitochondrial protein were resolved with SDS-PAGE (12% gel) and transferred to nitrocellulose membranes using Trans-Blot Turbo Midi Nitrocellulose Transfer Packs, and Trans-Blot Turbo Transfer Starter System (Bio-Rad Laboratories Inc., Hercules, CA, USA). After blocking with TBS containing 0.1% Tween (TBST) and 5% skim milk powder for 1 h at room temperature, the membranes were incubated overnight at 4°C with one of the following polyclonal antibodies: goat polyclonal anti-uncoupling protein 2 (UCP2), goat polyclonal anti-uncoupling protein 3 (UCP3) (both antibodies diluted at 1:1000) (Santa Cruz Biotechnology, Santa Cruz, CA, USA), or MitoProfile Total OXPHOS Rodent WB Antibody Cocktail (1:2000) (MitoSciences, Eugene, OR, USA) containing mouse monoclonal antibodies against Complex I-V subunits. Next, membranes were washed 3 × 5 min with TBST and incubated with a corresponding horse-radish peroxidase-conjugated secondary antibody for 1 h at room temperature. After the final wash of 3 × 5 min with TBST and 1 × 5 min with TBS, the immunocomplexes were detected using SuperSignal West Dura Extended Duration Substrate (Pierce, Thermo Fisher Scientific Inc., Rockford, IL, USA), visualized using ChemiDoc XRS+ imaging system and quantified using Image Lab analysis software version 3.0 (Bio-Rad Laboratories Inc., Hercules, CA, USA). Data were expressed relative to young controls.

### Statistical analysis

The data are expressed as averages ± SEM. The listed n values represent the number of mice used for a particular experiment. The statistical significance of the age and treatment (WBV) effects was assessed using two-way analysis of variance (ANOVA) with two between-subjects factors (age and WBV). Only if the interaction term between the factors was found to be significant, the effect of each factor was analyzed separately using Tukey post-hoc test. All analyses were performed with SPSS 20.0 (SPSS Inc., Chicago, IL, USA). The level of statistical significance was set at p<0.05.

## Results

### Animal characteristics

**Fig 1:**
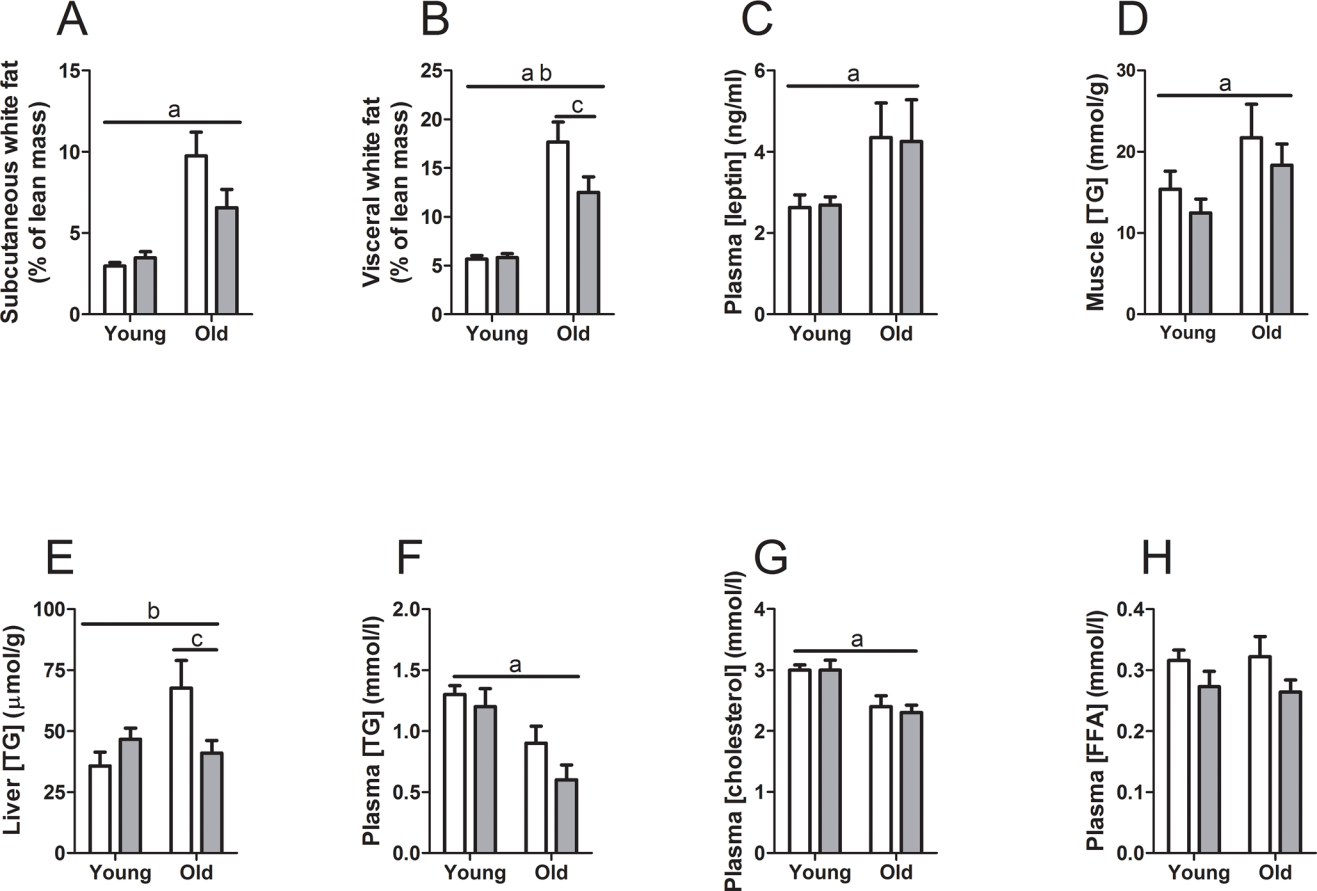
Animal characteristics. (A) subcutaneous white fat, (B) visceral white fat weights, (C) plasma leptin concentrations, (D) muscle triglyceride (TG) concentrations, (E) liver TG concentrations, (F) plasma TG concentrations, (G) plasma cholesterol concentrations, (H) plasma free fatty acid (FFA) concentrations. Data are averages from n = 7–9 mice per group; ± SEM. ^a^p<0.05, significant effect of age, ^b^p<0.05, interaction effect between age and WBV treatment, ^c^p<0.05, post-hoc treatment effect.

The effects of WBV on the animal characteristics at the end of the experimental protocol are summarized in Fig 1 and Table 1. Food intake was higher (F_1,39_ = 6.202; p<0.05) in old mice and this was not affected by WBV (Table 1). Body weight was significantly higher (F_1,39_ = 39.492; p<0.001) in old compared to young mice, with no significant effect of WBV (Table 1). The uncorrected dry lean mass was similar in all experimental groups (Table 1). For most examined organs and tissues, their dry lean mass-corrected weights were higher in old mice with no effect of WBV. The exception was the liver, where weights were lower in WBV treated mice irrespective of age (Table 1). The subcutaneous white (Fig 1A) and brown (Table 1) fat pad weights increased with age (respectively F_1,33_ = 27.805; p<0.001 and F_1,33_ = 5.193; p<0.05), with a strong tendency for significant interaction between age and WBV in the subcutaneous white fat weight (F_3,33_ = 28.48; p = 0.058). Further analysis revealed an effect of age on the visceral white fat deposits, which was treatment dependent (a significant interaction between age and WBV, F_3,33_ = 4.229; p<0.05) (Fig 1B). Visceral white fat pads were heavier in old control and WBV treated mice compared to corresponding young controls. WBV treatment reduced visceral white fat pads in old mice compared to old controls (F_1,15_ = 21.184; p<0.05), while in young mice WBV treatment had no effect. In agreement with the increase in white fat pad weight with age, we observed a significant (F_1,31_ = 6.382; p<0.05) increase in plasma leptin concentrations (Fig 1C), which correlated significantly with white fat pad weights in all groups (Pearson correlation coefficients: young control: 0.67, young WBV: 0.93, old control: 0.96 and old WBV: 0.96). Levels of triglycerides (TG) accumulated in skeletal muscle of old mice (F_1,32_ = 4.279; p<0.05), with no effect of WBV (Fig 1D). In liver, there was no main effect of age on TG levels, however, we did find a significant interaction between age and WBV treatment (F_3,33_ = 5.013; p<0.05), with significantly lower hepatic TG levels in WBV-treated old mice versus mice that did not undergo WBV treatment (F_1,16_ = 5.013; p<0.05). Thus, the effect of WBV treatment to lower TG concentrations was totally dependent on old age (Fig 1E). Plasma analyses revealed that TG and cholesterol concentrations were lower in old mice with no effect of WBV (Fig 1F and 1G). Plasma FFA concentrations were similar in young and old mice, while WBV treatment tended (F_1,32_ = 3.896; p = 0.058) to decrease plasma FFA concentrations (Fig 1H).

**Table 1:** Body composition. Average weekly food intake during eleven weeks of treatment, body weight at sacrifice, dry lean mass and organ weights (as percentage of dry lean mass). Data are means from n = 7–10 mice per group, ± SEM.

	Young		Old	
	Control	WBV	Control	WBV
**Food intake (kJ/wk)**	394.93±9.17	392.94±11.07	410.20±11.50^a^	425.06±14.23^a^
**Body weight (g)**	30.42±0.62	31.18±0.71	38.38±1.46^a^	35.21±1.72^a^
**Lean mass (g)**	27.86±0.60	28.33±0.51	29.81±0.60	29.25±0.92
**Heart (%)**	0.49±0.00	0.48±0.01	0.56±0.03^a^	0.54±0.02^a^
**Spleen (%)**	0.30±0.02	0.32±0.03	0.29±0.02	0.30±0.04
**Seminal vesicles (%)**	0.95±0.03	0.97±0.03	3.60±0.76^a^	3.56±1.11^a^
**Testes (%)**	0.79±0.01	0.75±0.01	0.65±0.02^a^	0.62±0.02^a^
**Brown fat (%)**	0.57±0.02	0.69±0.03	1.21±0.23^a^	0.99±0.40^a^
**Liver (%)**	5.86±0.13	5.65±0.13^b^	6.33±0.23	5.63±0.33^b^

^a^p<0.05, significant effect of age.

^b^p<0.05, significant effect of WBV treatment.

### Indirect calorimetry

**Fig 2:**
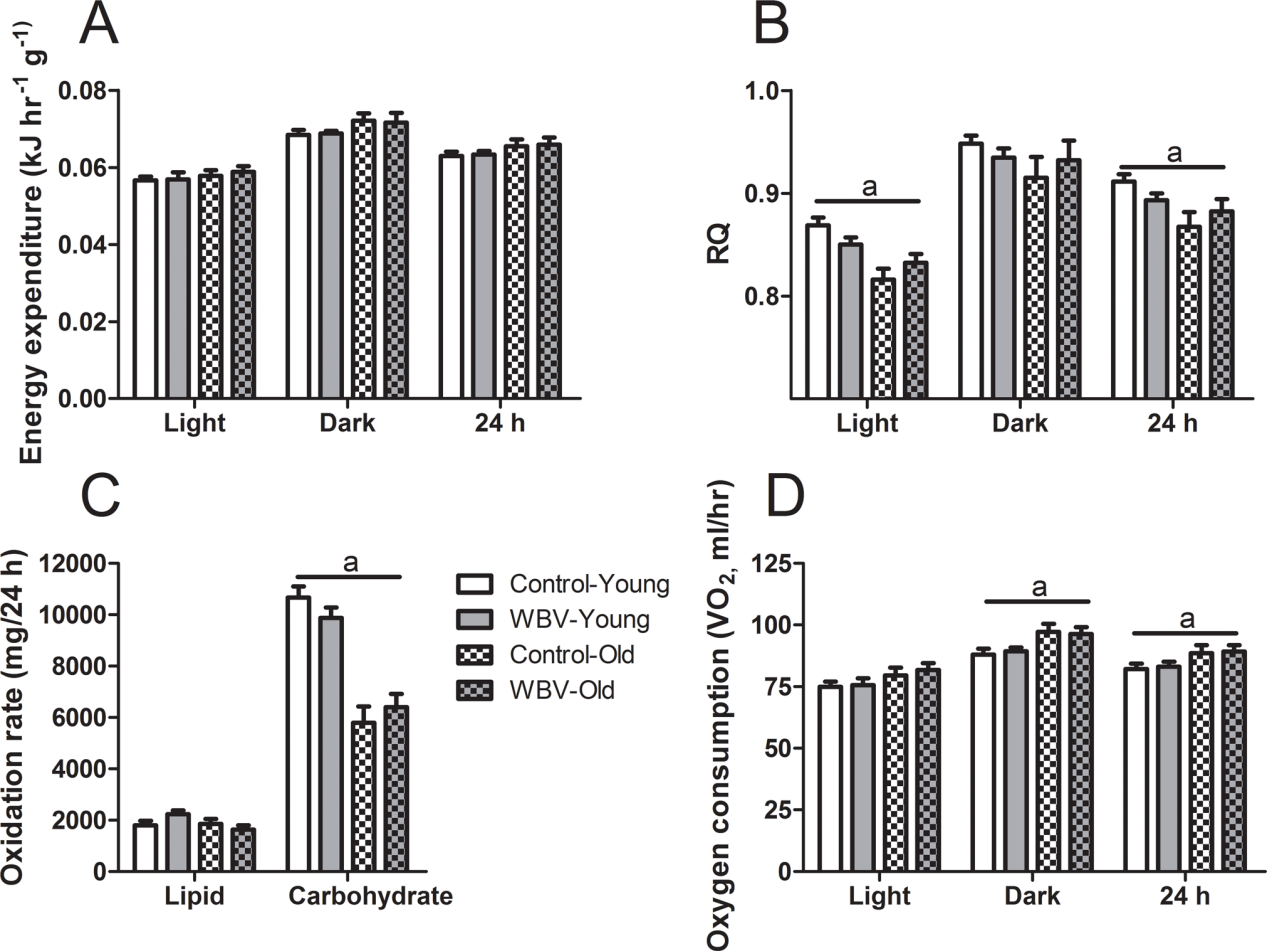
Energy expenditure and substrate utilization *in vivo* after nine weeks of WBV treatment. (A) Average dry lean mass-corrected energy expenditures (EE) and (B) average respiratory quotients (RQ) during light phase, dark phase and 24 hours. (C) Average lipid and carbohydrate oxidation rates per 24 hours. (D) Average oxygen consumption during light phase, dark phase and 24 hours. Data are averages from n = 7–8 mice per group; ± SEM. ^a^ p<0.05, significant effect of age.

Fig 2 illustrates energy expenditure (EE) and substrate utilization data *in vivo*. The lean mass-corrected EE was similar in all experimental groups (Fig 2A), but it was significantly lower in old mice when corrected for total body weight (S1 Fig). The average respiratory quotient (RQ) during 24 hours of measurement was lower in old compared to young mice (F_1,30_ = 6.840; p<0.05), indicating decreased utilization of carbohydrates over fats as energy source (Fig 2B). The observed difference was the consequence of lower average RQ in the light phase (F_1,30_ = 5.013; p<0.05) with no differences in the dark phase. Further analysis revealed a major decrease in carbohydrate oxidation in response to aging (F_1,30_ = 67.223; p<0.001) with no effect on lipid oxidation (Fig 2C). WBV treatment had no effect on carbohydrate or lipid oxidation rates.

### Blood glucose turnover and hepatic carbohydrate fluxes

**Fig 3:**
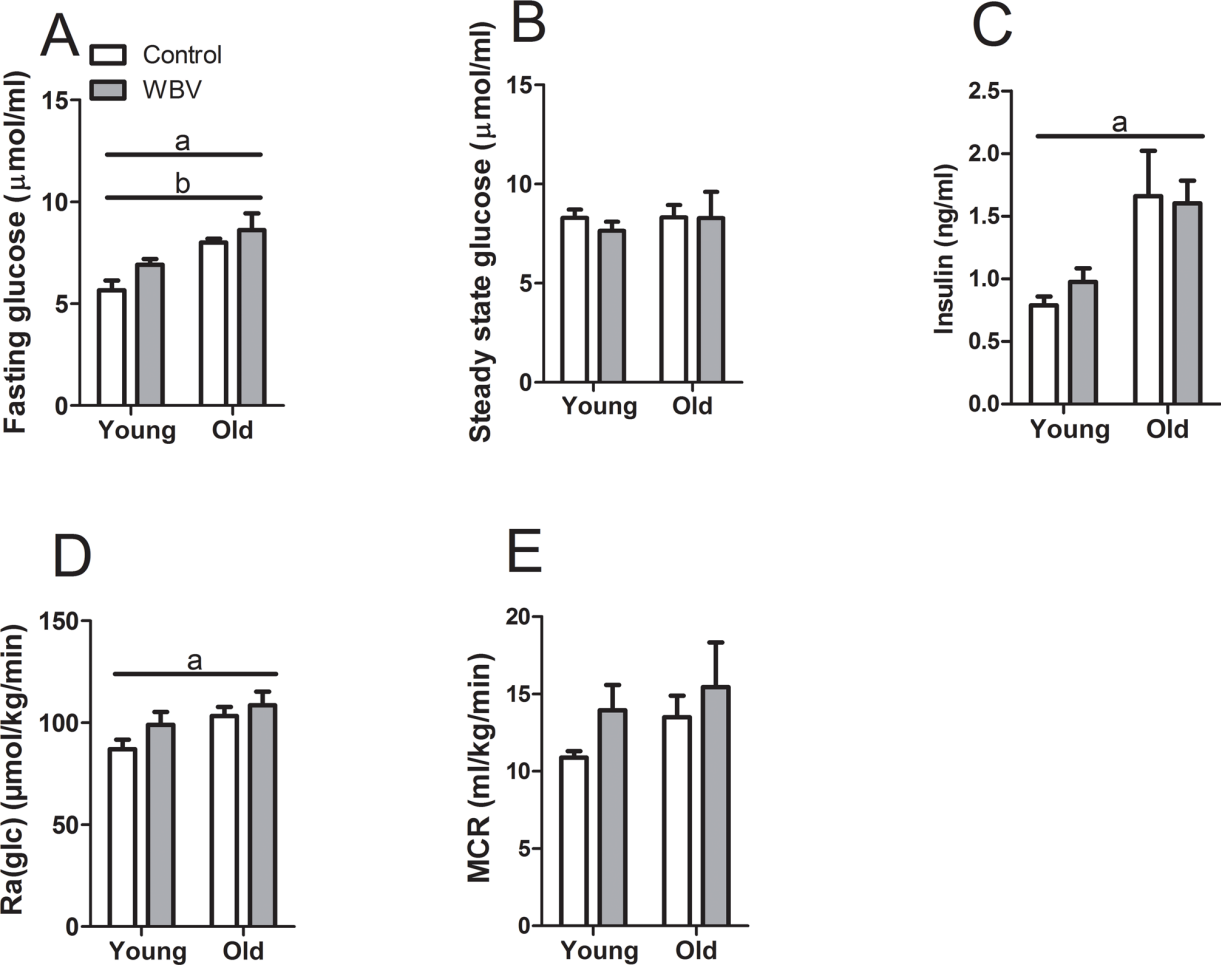
Blood glucose turnover rates after 12–13 weeks of WBV treatment. (A) Initial (fasting) and (B) steady-state glucose concentrations during stable isotope infusion experiment. (C) Initial (fasting) insulin concentrations. (D) Endogenous glucose production rates (Ra(glc)). (E) Metabolic clearance rates of blood glucose (MCR), calculated as the ratios of total glucose turnover rates and blood glucose concentrations. Data are averages from n = 6–9 mice per group; ± SEM. ^a^p<0.05, significant effect of age; ^b^ p<0.05, significant of WBV.

To investigate whether the reduced carbohydrate utilization was reflected in regulation of glucose metabolism, stable isotope infusion experiments were performed in conscious mice. Both aging (F_1,30_ = 19.610; p<0.001) and WBV treatment (F_1,30_ = 4.159; p = 0.05) led to an increase in fasting blood glucose concentrations (measured before starting the infusion) (Fig 3A), while steady-state blood glucose concentrations during the infusion were similar in all experimental groups (Fig 3B). Initial (fasting) insulin concentrations were increased in old mice (F_1,38_ = 9.444; p<0.01) (Fig 3C), with no effect by WBV. Steady-state endogenous glucose appearance rates (Ra(glc)) (Fig 3D) and glucose disposal rates (Rd(glc)) (S2 Fig) increased with age (respectively F_1,26_ = 5.410 and F_1,26_ = 4.823; p<0.05), an outcome that seemingly contrasted with our expectation based on the indirect calorimetry data. WBV treatment tended to increase both glucose appearance (F_1,26_ = 2.426; p = 0.13) and disposal rates(F_1,26_ = 2.440; p = 0.13). Metabolic clearance rates of blood glucose (MCR = Rd(glc) / [glucose]) were not affected by age, but tended to increase (F_1,26_ = 2.207; p = 0.149) in response to WBV treatment (Fig 3E).

**Fig 4:**
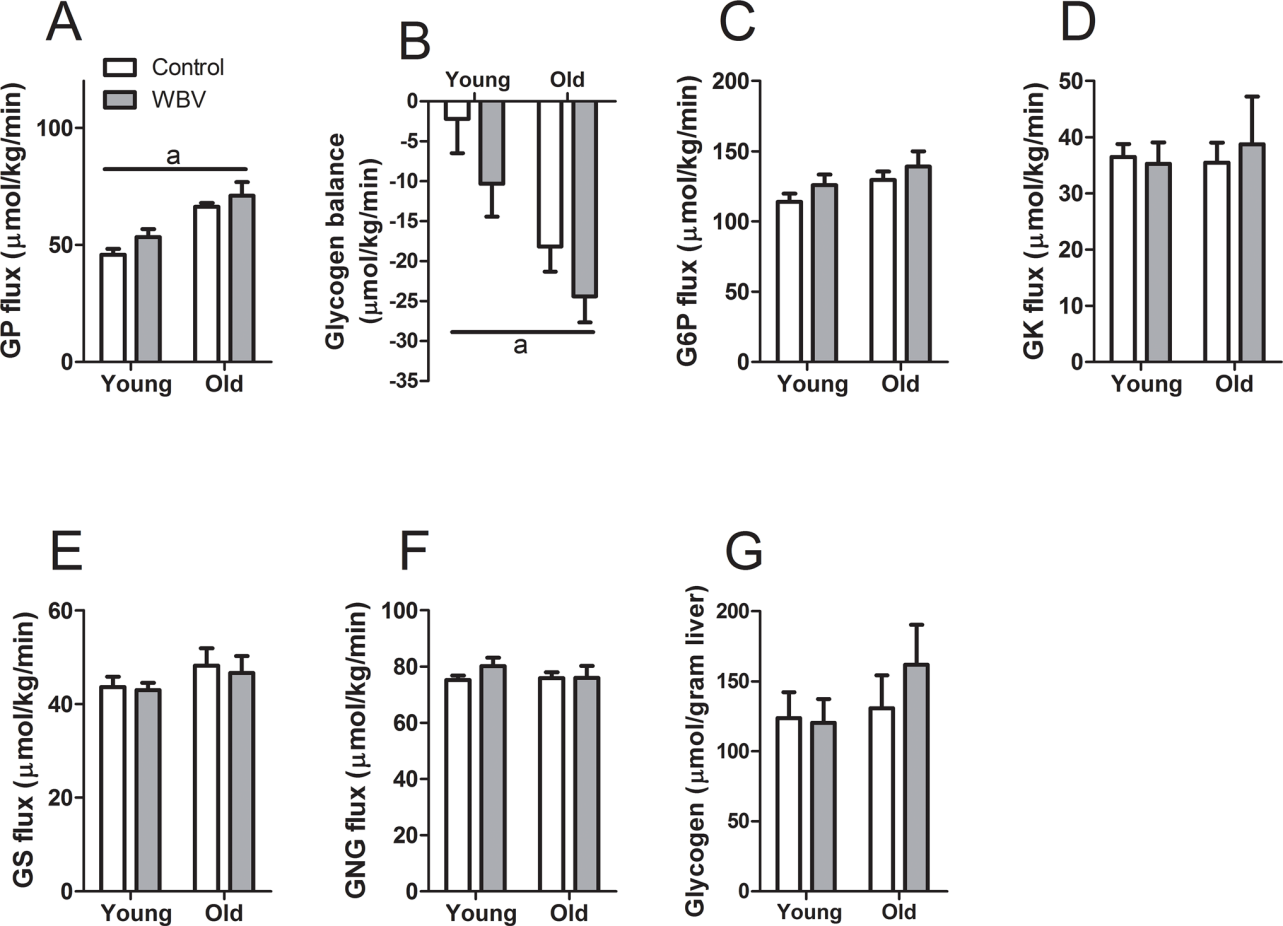
Hepatic carbohydrate fluxes *in vivo* after 12–13 weeks of WBV treatment. (A) Glycogen phosphorylase (GP) flux. (B) Glycogen balance, calculated by subtracting GP flux from glycogen synthase (GS) flux. (C) Glucose-6-phosphatase (G6P) flux. (D) Glucokinase (GK) flux. (E) GS flux. (F) Total gluconeogenesis (GNG) flux. (G) Hepatic glycogen content. Data are averages from n = 6–9 mice per group; ± SEM. ^a^p<0.05, significant effect of age.

Next we performed a detailed analysis of hepatic carbohydrates fluxes *in vivo* to identify possible causes of the increase in Ra(glc) (Fig 4). Except for the flux associated with glycogen phosphorylase (GP) (F_1,26_ = 33.690; p <0.001), no significant changes were observed in the fluxes associated with glucose-6-phosphatase (G6P), glucokinase (GK), glycogen synthase (GS) and gluconeogenesis (GNG) (Fig 4C–4F). WBV treatment tended to increase the GP flux (F_1,26_ = 3.408; p = 0.076) resulting in a tendency of increasingly negative glycogen balance (F_1,26_ = 3.536; p = 0.071) (Fig 4A and 4B respectively). It should be realized, however, that in the applied isotopic model of hepatic carbohydrate metabolism the GP flux is used to balance all hepatic carbohydrate fluxes to account for the Ra(glc) and is not measured directly. Alternatively, the increased Ra(glc) may be due to sources of glucose production other than the liver.

### Effects of WBV on the mitochondrial properties in liver and skeletal muscle

**Fig 5:**
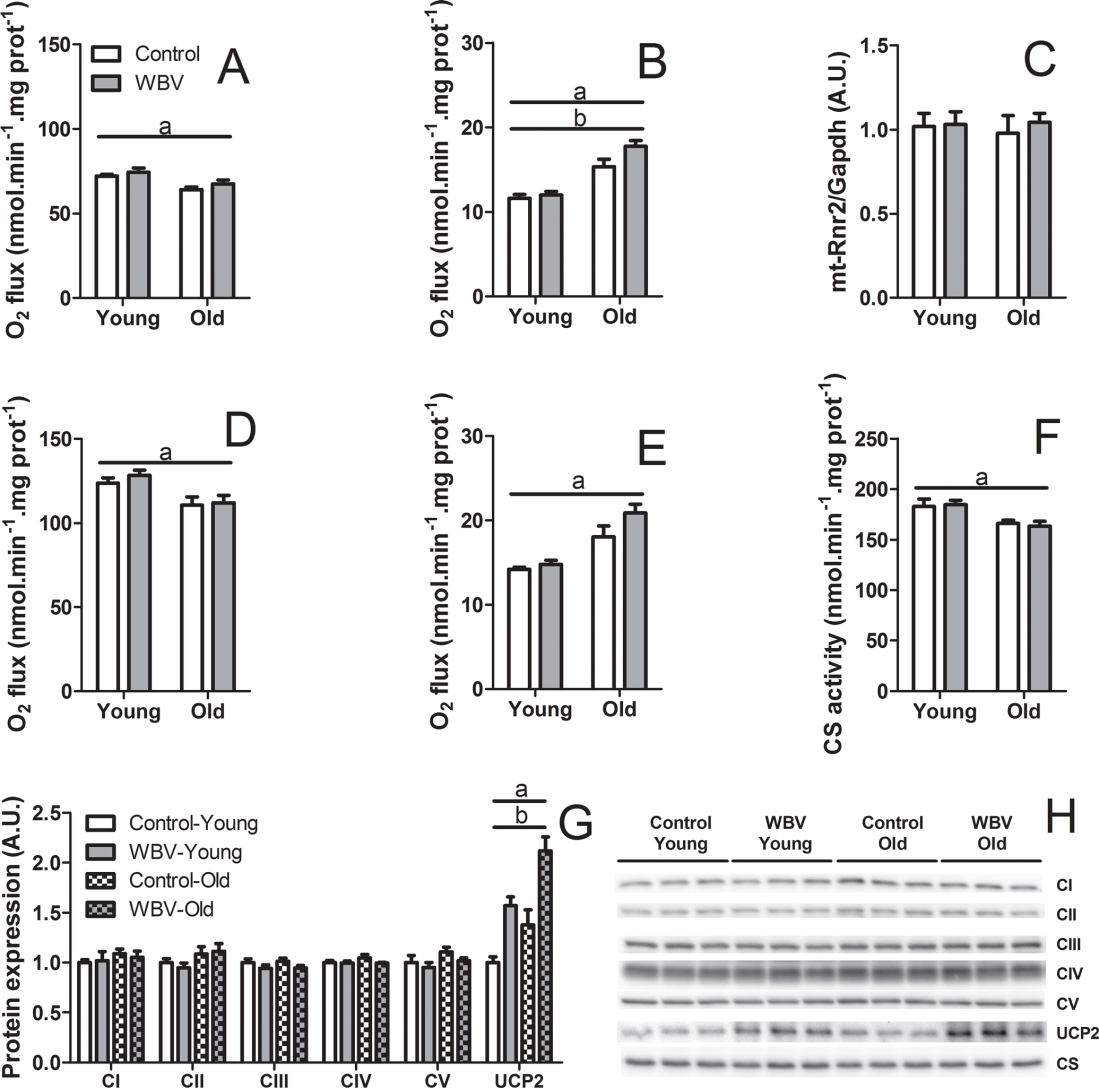
Effects of 14 weeks of WBV treatment on the mitochondrial properties in livers of young and old mice. (A) Maximal ADP-stimulated O_2_ flux (state 3) and (B) basal O_2_ flux (state 4) in isolated liver mitochondria oxidizing pyruvate plus malate. (C) Relative mtDNA copy number in liver. (D) Maximal ADP-stimulated O_2_ flux (state 3) and (E) basal O_2_ flux (state 4) in isolated liver mitochondria oxidizing palmitoyl-CoA plus L-carnitine plus malate. (F) Citrate synthase (CS) activity in liver. (G) Relative protein levels and (H) representative immunoblot images of selected subunits of oxidative phosphorylation pathway complexes I-V and uncoupling protein 2 (UCP2) in isolated liver mitochondria. Data are means from n = 6–9 (A-F) or n = 3 (G-H) mice per group; ± SEM. ^a^ p<0.05, significant effect of age. ^b^p<0.05, significant effect of WBV.

To examine the mechanism underlying the reduction of white fat mass and plasma TG in response to WBV in old mice, we analyzed the function of isolated mitochondria from liver and skeletal muscle by high-resolution respirometry. Maximal ADP-stimulated O_2_ flux (state 3) in isolated liver mitochondria oxidizing tricarboxylic acid (TCA) cycle substrates pyruvate plus malate decreased significantly (F_1,32_ = 14.454; p<0.01) with age, without an effect by WBV (Fig 5A). Both aging and WBV led to an increase (F_1,32_ = 53.101; p<0.001 and F_1,32_ = 4.764; p<0.05, respectively) in basal O_2_ flux (state 4), which was measured in the absence of ATP synthesis (Fig 5B). The WBV effect tended to be stronger in old mice (16% increase) than in young mice (5% increase) (Fig 5B). This suggests increased proton permeability of the inner mitochondrial membrane, since basal O_2_ flux is stimulated by backflow of protons into mitochondrial matrix. The same trends were observed in isolated liver mitochondria oxidizing the fatty acid β-oxidation substrate palmitoyl-CoA (Fig 5D and 5E). The protein levels of selected subunits of oxidative phosphorylation complexes in isolated liver mitochondria were not affected by aging or WBV (Fig 5G and 5H). In contrast, the protein level of UCP2 was strongly increased in response to both aging (F_1,11_ = 16.337; p<0.01) and WBV (F_1,11_ = 32.546; p<0.01). Clearly, the UCP2 levels were far highest in the old mice that underwent WBV (Fig 5G and 5H), and it is therefore somewhat surprising that the differences in basal O_2_ fluxes were not more pronounced in the old mice. Finally, we measured relative mtDNA copy number and the activity of the mitochondrial enzyme citrate synthase as markers of mitochondrial density. The relative mtDNA copy number was not affected by aging or WBV (Fig 5C). The activity of citrate synthase decreased significantly (F_1,29_ = 12.590; p<0.01) in response to aging without an effect by WBV (Fig 5F).

**Fig 6:**
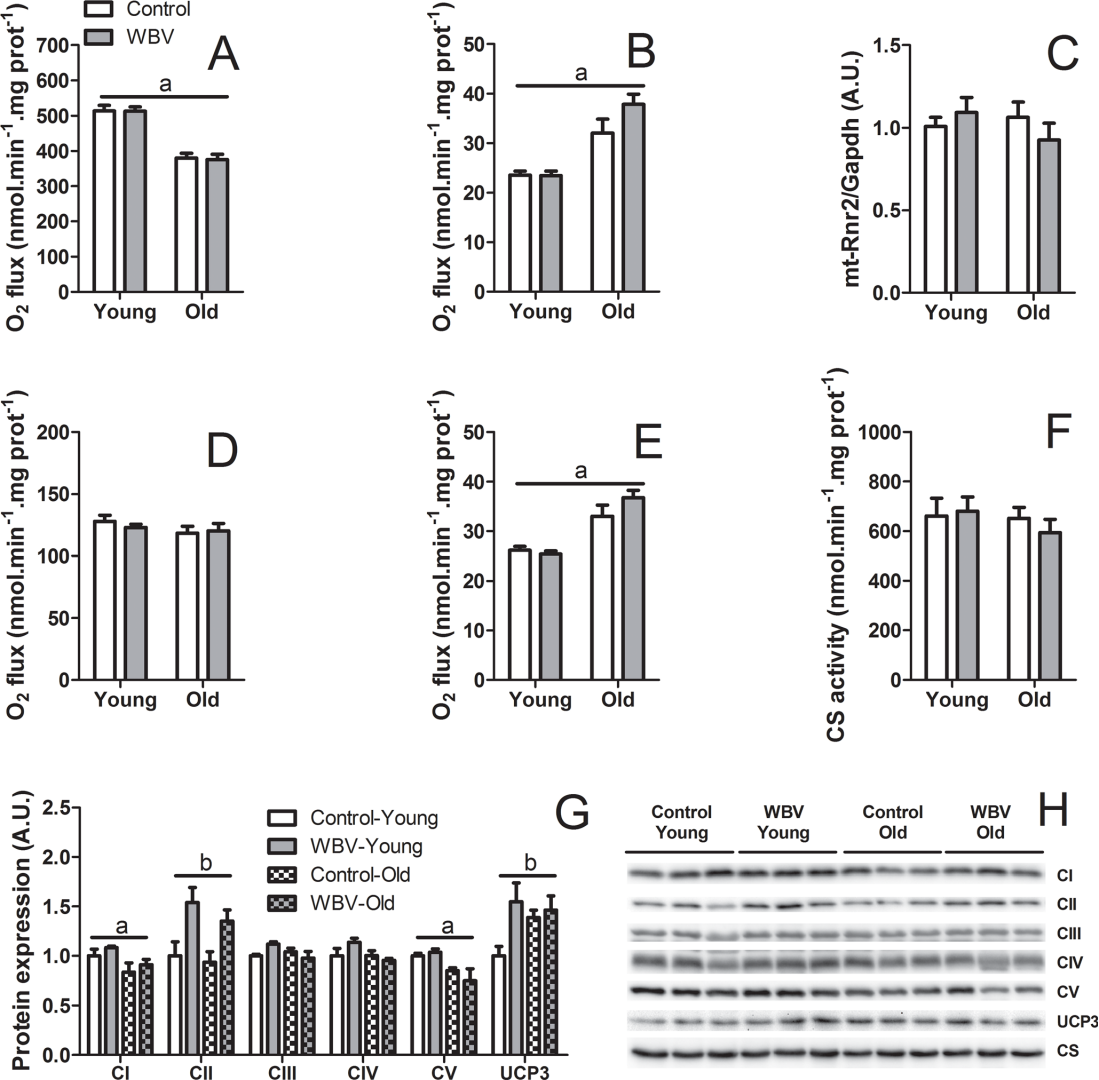
Effects of 14 weeks of WBV treatment on the mitochondrial properties in skeletal muscle of young and old mice. (A) Maximal ADP-stimulated O_2_ flux (state 3) and (B) basal O_2_ flux (state 4) in isolated skeletal muscle mitochondria oxidizing pyruvate plus malate. (C) Relative mtDNA copy number in skeletal muscle. (D) Maximal ADP-stimulated O_2_ flux (state 3) and (E) basal O_2_ flux (state 4) in isolated skeletal muscle mitochondria oxidizing palmitoyl-CoA plus L-carnitine plus malate. (F) Citrate synthase (CS) activity in skeletal muscle. (G) Relative protein levels and (H) representative immunoblot images of selected subunits of oxidative phosphorylation pathway complexes I-V and uncoupling protein 3 (UCP3) in isolated skeletal muscle mitochondria. Data are means from n = 6–9 (A-F) or n = 3 (G-H) mice per group; ± SEM. ^a^p<0.05, significant effect of age; ^b^p<0.05, significant effect of WBV.

Fig 6 shows the same analysis in skeletal muscle mitochondria, yielding similar results as in the liver. When oxidizing pyruvate plus malate, muscle mitochondria showed a more pronounced decline of maximal ADP-stimulated O_2_ flux in response to aging compared to liver mitochondria, without an effect by WBV (Fig 6A). The decline was accompanied by lower protein levels of the oxidative phosphorylation Complexes I and V subunits (*i*.*e*. NADH dehydrogenase and ATP synthase) (Fig 6G and 6H). Interestingly, WBV caused an upregulation of the Complex II subunit, albeit without apparent consequences on O_2_ fluxes. The maximal ADP-stimulated O_2_ flux with palmitoyl-CoA as the oxidizable substrate was neither affected by aging nor by WBV (Fig 6D). As in liver mitochondria, the basal O_2_ flux increased with age with both TCA cycle and fatty acid β-oxidation substrates (Fig 6B–6E), without an effect of WBV. Interestingly, WBV increased protein levels of UCP3 (F_1,11_ = 5.383; p<0.05) an UCP isoform typical to skeletal muscle [38], but this effect was most pronounced in young mice. This pattern is somewhat different as the hepatic UCP2 pattern in which the effect of WBV to upregulate UCP2 levels was equal in both young and old mice. The assessment of mtDNA copy number (Fig 6C) and citrate synthase activity (Fig 6F) in skeletal muscle revealed no changes in response to aging or WBV.

## Discussion

WBV training is gaining interest as an effective alternative to physical activity in frail population groups. Indeed, it has been shown that in older adults WBV was effective in increasing muscle strength [17,39–42] and bone mineral density [17,43]. In the present study we performed a detailed characterization of energy balance parameters as well as *in vivo* and *ex vivo* metabolic parameters in young and old mice, and, subsequently investigated the effects of WBV for ten minutes a day, five days a week for 8–14 weeks on these parameters. Most notably, in old -but not young- mice WBV treatment significantly decreased visceral fat mass and triglyceride content in the liver, which was associated with an upregulation of mitochondrial UCP2 and mitochondrial proton leak. Collectively, these observations suggest that WBV stimulates hepatic lipid oxidation and alleviates the negative effects of fatty liver in old mice. Indeed, liver TGs were selectively reduced by WBV in old mice (Fig 1G).

In agreement with data in old mice [44] and humans [45], we showed that carbohydrate utilization (inferred by whole-body indirect calorimetry) decreased with age. These data are in line with elevated fasting plasma concentrations of glucose and insulin in old mice (i.e., markers of impaired glucose homeostasis). Since WBV treatment resulted in a reduction of visceral adiposity in old mice, we expected a beneficial effect of WBV on above-mentioned whole-body metabolic parameters. However, WBV treatment did not affect these parameters, indicating no sustained changes in whole-body metabolic parameters exceeding beyond those that probably occurred during WBV as a direct response to the exercise load.

It has been shown that an increase in adiposity during aging is related to lipid accumulation in various non-adipose tissues in rats [46], and this was confirmed by the increased TG content in response to aging in both liver and skeletal muscle. WBV treatment resulted in a strong reduction of TG content in the liver of old but not young mice, which is in line with the reduction of white fat mass in old WBV-treated mice. The lack of a pronounced effect of WBV on the TG content in skeletal muscle may be explained by the fact that trained muscle actually tends to store TG [47], which can be used to fuel contractions in particular during low intensity physical activity. The tissue TG content can be modulated by the mitochondrial capacity to oxidize fatty acids and by mitochondrial density. The mtDNA copy number and citrate synthase activity were hardly affected by aging or WBV in both skeletal muscle and liver, suggesting unaltered mitochondrial density. Unaltered mtDNA copy number was observed in liver and skeletal muscle of C57BL/6J mice at a comparable old age as in our study [44], while lower mtDNA copy number was reported in rat liver and skeletal muscle at older age (27 months) [48] and rat skeletal muscle at a comparable age (22 months) [49] as in our study. These data suggest that the onset of the aging-induced decline of the mitochondrial density may vary depending on the species and experimental conditions. The fact that citrate synthase activity was lower in livers of old compared to young mice without a notable decline in mtDNA copy number may indicate that decline in enzyme activity preceded decline in the mitochondrial density. Houtkooper and colleagues [44] showed that the expression of genes involved in fatty-acid oxidation decrease with age in mouse liver. In line with this we showed at the functional level that mitochondrial fatty-acid oxidation capacity decreased in isolated liver mitochondria from old compared to young mice. This contributes to a lower tissue capacity to oxidize fatty acids. The capacity to oxidize the glucose-derived substrate pyruvate was strongly decreased in skeletal muscle mitochondria of old compared to young mice too, with only a slight reduction observed in liver mitochondria, indicating decreased oxidative glucose utilization in particular in skeletal muscle.

In agreement with published data [46], the impairment of skeletal muscle mitochondrial function was related to down-regulation of oxidative phosphorylation pathway complex I and V. Decreased pyruvate oxidation capacity is in agreement with our *in vivo* indirect calorimetry results showing reduced carbohydrate utilization in old mice. These data seemingly contradict with the results of the stable-isotope-infusion experiments showing increased rates of glucose disposal in old mice. However, indirect calorimetry assesses only complete aerobic carbohydrate oxidation, while the stable-isotope experiment yielded glucose disposal independent of the mode of metabolism (*e*.*g*. glycolytic versus oxidative). One explanation pertinent to these data may be that aging promotes glycolysis to compensate for a diminished mitochondrial capacity to oxidize pyruvate; *i*.*e*. the so-called Warburg effect, in aging skeletal muscle [50]. This is in agreement with elevated lactate levels in tissues of aged mice [44].

We showed that WBV had no effect on either fatty acid or pyruvate oxidation in the coupled state (state 3) in both liver and skeletal muscle mitochondria. Similar findings were reported in young WBV-treated rats, showing unaltered O_2_ consumption rates in permeabilized skeletal muscle fibers [50]. In agreement with Andrews and colleagues [51], the basal O_2_ consumption rates (state 4) were increased in response to aging in both liver and skeletal muscle mitochondria, suggesting increased leakiness of the inner mitochondrial membrane for protons. WBV treatment augmented the basal O_2_ consumption rates in particular in old mice. The increase in basal respiration was associated with upregulation of UCP2 in liver mitochondria and UCP3 in skeletal muscle mitochondria (albeit the latter only in the young mice). While upregulation of UCP2 and UCP3 in tissues of old rats has been reported previously [52], our data are the first to show that their expression is affected by WBV. The precise physiological functions of UCPs in non-adipose tissues remain to be established and new functions of UCPs as substrate transporters are emerging [53]. It has been suggested that mild uncoupling of mitochondrial oxidative phosphorylation can act as a protective mechanism to reduce the production of the reactive oxygen species by the mitochondrial respiratory chain [54,55]. Dissipation of energy through uncoupling may contribute to the reduction of tissue TG content [56]. An acute effect of WBV is the activation of the sympathetic nervous system [57]. Catecholamines, which are released upon this activation, have been shown to up-regulate UCP2 and UCP3 expression in L6 myotubes [58]. Moreover, data in literature show that UCPs are often upregulated under conditions of increased fatty acid availability [59,60], suggesting that these proteins indeed play a role in regulation of lipid metabolism. The postulated requirement of fatty acids for stimulation of uncoupling activity of UCPs [55,61] may explain why upregulation of UCPs in the tissues of young animals, which contain less fat, does not elicit the TG-reducing effect as observed in the tissues of old animals.

In summary, we showed that chronic WBV treatment was effective in reducing adiposity in old but not young mice. Changes in body composition induced by WBV were associated with a reduction of liver TG stores, and increased mitochondrial uncoupling in the liver, suggesting that WBV stimulates hepatic lipid metabolism. These alterations in fuel fluxes were independent of alterations in energy balance parameters such as food intake and/or whole-body energy expenditure. We speculate that there are different temporal effects of WBV (e.g. sympathetic activity) and sustained for others (e.g. increased hepatic UCP2 expression and mitochondrial oxidation, reversed hepatic hypertriglyceridemia). Because the latter changes occurred independent of changes in food intake and whole-body metabolic rate (assessed by indirect calorimetry), the liver-specific effects of WBV may be a primary mechanism to improve metabolic health during aging, rather than that it is a consequence of alterations in energy balance.

## Supporting Information

S1 Fig Energy expenditures corrected for body weight. Data are averages from n = 7–8 mice per group; ± SEM. ^a^p<0.05, significant effect of age.
(TIF)

S2 Fig Steady-state glucose disposal rates (Rd(glc)). Data are averages from n = 6–9 mice per group; ± SEM. ^a^p<0.05, significant effect of age.
(TIF)

## References

[1] United Nations. Department of Economic and Social Affairs, 2013. Population Division. 2013. doi:ST/ESA/SER.A/348

[2] Kim TN, Choi KM. The implications of sarcopenia and sarcopenic obesity on cardiometabolic disease. J Cell Biochem. 2014; 10.1002/jcb.25077 25545054

[3] Fielding RA, Vellas B, Evans WJ, Bhasin S, Morley JE, Newman AB, et al Sarcopenia: An Undiagnosed Condition in Older Adults. Current Consensus Definition: Prevalence, Etiology, and Consequences. International Working Group on Sarcopenia. J Am Med Dir Assoc. Elsevier Ltd; 2011;12: 249–256. 10.1016/j.jamda.2011.01.003 21527165 PMC3377163

[4] Liu C-J, Latham NK. Progressive resistance strength training for improving physical function in older adults. Cochrane Database Syst Rev. 2009;3 10.1002/14651858.CD002759.pub2 PMC4324332 19588334

[5] Bann D, Hire D, Manini T, Cooper R, Botoseneanu A, McDermott MM, et al Light Intensity Physical Activity and Sedentary Behavior in Relation to Body Mass Index and Grip Strength in Older Adults: Cross-Sectional Findings from the Lifestyle Interventions and Independence for Elders (LIFE) Study. PLoS One. 2015;10: e0116058 10.1371/journal.pone.0116058 25647685 PMC4315494

[6] Conn VS, Koopman RJ, Ruppar TM, Phillips LJ, Mehr DR, Hafdahl AR. Insulin Sensitivity Following Exercise Interventions: Systematic Review and Meta-Analysis of Outcomes Among Healthy Adults. J Prim Care Community Health. 2014;5: 211–222. 10.1177/2150131913520328 24474665 PMC4393364

[7] Issurin VB, Tenenbaum G. Acute and residual effects of vibratory stimulation on explosive strength in elite and amateur athletes. J Sports Sci. 1999;17: 177–182. 10.1080/026404199366073 10362384

[8] Lau RW, Liao L-R, Yu F, Teo T, Chung RC, Pang MY. The effects of whole body vibration therapy on bone mineral density and leg muscle strength in older adults: a systematic review and meta-analysis. Clin Rehabil. 2011;25: 975–988. 10.1177/0269215511405078 21849376

[9] Bosco C, Colli R, Introini E, Cardinale M, Tsarpela O, Madella a, et al Adaptive responses of human skeletal muscle to vibration exposure. Clin Physiol. 1999;19: 183–187. 10.1046/j.1365-2281.1999.00155.x 10200901

[10] Burke D, Schiller HH. Discharge pattern of single motor units in the tonic vibration reflex of human triceps surae. J Neurol Neurosurg Psychiatry. 1976;39: 729–741. 10.1136/jnnp.39.8.729 956859 PMC492438

[11] Prisby RD, Lafage-Proust M-H, Malaval L, Belli A, Vico L. Effects of whole body vibration on the skeleton and other organ systems in man and animal models: what we know and what we need to know. Ageing Res Rev. 2008;7: 319–29. 10.1016/j.arr.2008.07.004 18762281

[12] Hazell TJ, Lemon PWR. Synchronous whole-body vibration increases VO_2_ during and following acute exercise. Eur J Appl Physiol. 2012;112: 413–20. 10.1007/s00421-011-1984-2 21573780

[13] Bosco C, Iacovelli M, Tsarpela O, Cardinale M, Bonifazi M, Tihanyi J, et al Hormonal responses to whole-body vibration in men. Eur J Appl Physiol. 2000;81: 449–54. 10.1007/s004210050067 10774867

[14] Kvorning T, Bagger M, Caserotti P, Madsen K. Effects of vibration and resistance training on neuromuscular and hormonal measures. Eur J Appl Physiol. 2006;96: 615–25. 10.1007/s00421-006-0139-3 16482475

[15] Rittweger J, Ehrig J, Just K, Mutschelknauss M, Kirsch K a, Felsenberg D. Oxygen uptake in whole-body vibration exercise: influence of vibration frequency, amplitude, and external load. Int J Sports Med. 2002;23: 428–32. 10.1055/s-2002-33739 12215962

[16] Vissers D, Verrijken A, Mertens I, Van Gils C, Van de Sompel A, Truijen S, et al Effect of long-term whole body vibration training on visceral adipose tissue: a preliminary report. Obes Facts. 2010;3: 93–100. 10.1159/000301785 20484941 PMC6452127

[17] Verschueren SMP, Roelants M, Delecluse C, Swinnen S, Vanderschueren D, Boonen S. Effect of 6-month whole body vibration training on hip density, muscle strength, and postural control in postmenopausal women: a randomized controlled pilot study. J Bone Miner Res. 2004;19: 352–9. 10.1359/JBMR.0301245 15040822

[18] Baum K, Votteler T, Schiab J. Efficiency of vibration exercise for glycemic control in type 2 diabetes patients. Int J Med Sci. 2007;4: 159–63. 17554399 10.7150/ijms.4.159 PMC1885552

[19] Vissers D, Baeyens J-P, Truijen S, Ides K, Vercruysse C-C, Van Gaal L. The effect of whole body vibration short-term exercises on respiratory gas exchange in overweight and obese women. Phys Sportsmed. 2009;37: 88–94. 10.3810/psm.2009.10.1733 20048532

[20] Cochrane DJ. Is vibration exercise a useful addition to a weight management program? Scand J Med Sci Sports. 2012;22: 705–13. 10.1111/j.1600-0838.2011.01411.x 22092513

[21] Wilms B, Frick J, Ernst B, Mueller R, Wirth B, Schultes B. Whole body vibration added to endurance training in obese women—a pilot study. Int J Sports Med. 2012;33: 740–3. 10.1055/s-0032-1306284 22562734

[22] Roelants M, Delecluse C, Goris M, Verschueren S. Effects of 24 weeks of whole body vibration training on body composition and muscle strength in untrained females. Int J Sports Med. 2004;25: 1–5. 10.1055/s-2003-45238 14750005

[23] Naghii MR, Ghanizadeh G, Darvishi P, Ebrahimpour Y, Mofid M, Torkaman G, et al Whole body vibration is a safe exercise training method and induces no impaired alterations on rat plasma parameters. Acta Physiol Hung. 2011;98: 442–8. 10.1556/APhysiol.98.2011.4.7 22173025

[24] Maddalozzo GF, Iwaniec UT, Turner RT, Rosen CJ, Widrick JJ. Whole-body vibration slows the acquisition of fat in mature female rats. Int J Obes (Lond). 2008;32: 1348–54. 10.1038/ijo.2008.111 18663370 PMC2586051

[25] Regterschot GRH, Van Heuvelen MJG, Zeinstra EB, Fuermaier ABM, Tucha L, Koerts J, et al Whole body vibration improves cognition in healthy young adults. PLoS One. 2014;9 10.1371/journal.pone.0100506 PMC4065066 24949870

[26] Timmer M, Van Der Zee EA, Riedel G. Whole body vibration and behaviour: Investigation of the role of various neurotransmitter systems. FENS Forum Abstr. 2006;3.

[27] Van Der Zee EA, Riedel G, Rutgers EH, de Vries C, Postema F, Venema BJ, et al Enhanced neuronal activity in selective brain regions of mice induced by whole body stimulation. FENS Forum Abstr. 2010;5.

[28] Oklejewicz M, Hut RA, Daan S, Loudon AS, Stirland AJ. Metabolic rate changes proportionally to circadian frequency in tau mutant Syrian hamsters. J Biol Rhythms. 1997;12: 413–22. 9376640 10.1177/074873049701200503

[29] Ferrannini E. The theoretical bases of indirect calorimetry: a review. Metabolism. 1988;37: 287–301. 10.1016/0026-0495(88)90110-2 3278194

[30] Lusk G. The elements of the science of nutrition Johnson Reprint Corporation, New York; 1976.

[31] Van Dijk TH, Boer TS, Havinga R, Stellaard F, Kuipers F, Reijngoud D-J. Quantification of hepatic carbohydrate metabolism in conscious mice using serial blood and urine spots. Anal Biochem. 2003;322: 1–13. 10.1016/j.ab.2003.07.008 14705774

[32] Van Dijk TH, van der Sluijs FH, Wiegman CH, Baller JFW, Gustafson L a, Burger HJ, et al Acute inhibition of hepatic glucose-6-phosphatase does not affect gluconeogenesis but directs gluconeogenic flux toward glycogen in fasted rats. A pharmacological study with the chlorogenic acid derivative S4048. J Biol Chem. 2001;276: 25727–35. 10.1074/jbc.M101223200 11346646

[33] Van den Broek NMA, Ciapaite J, De Feyter HMML, Houten SM, Wanders RJA, Jeneson JAL, et al Increased mitochondrial content rescues in vivo muscle oxidative capacity in long-term high-fat-diet-fed rats. FASEB J. 2010;24: 1354–64. 10.1096/fj.09-143842 20040520

[34] Mildaziene V, Nauciene Z, Baniene R, Grigiene J. Multiple effects of 2,2’,5,5'-tetrachlorobiphenyl on oxidative phosphorylation in rat liver mitochondria. Toxicol Sci. 2002;65: 220–7. 11812926 10.1093/toxsci/65.2.220

[35] Bligh EG, Dyer WJ. A rapid method of total lipid extraction and purification. Can J Biochem Physiol. 1959;37: 911–7. 13671378 10.1139/o59-099

[36] Lavoinne a, Baquet a, Hue L. Stimulation of glycogen synthesis and lipogenesis by glutamine in isolated rat hepatocytes. Biochem J. 1987;248: 429–437. 3124812 10.1042/bj2480429 PMC1148559

[37] Srere PA, Brazil H, Gonen L. The citrate condensing enzyme of pigeon breast muscle and moth flight muscle. Acta Chem Scand. 1963;

[38] Vidal-Puig A, Solanes G, Grujic D, Flier JS, Lowell BB. UCP3: an uncoupling protein homologue expressed preferentially and abundantly in skeletal muscle and brown adipose tissue. Biochem Biophys Res Commun. 1997;235: 79–82. 10.1006/bbrc.1997.6740 9196039

[39] Rees SS, Murphy AJ, Watsford ML. Effects of whole-body vibration exercise on lower-extremity muscle strength and power in an older population: a randomized clinical trial. Phys Ther. 2008;88: 462–470. 10.2522/ptj.20070027 18218826

[40] Tapp LR, Signorile JF. Efficacy of WBV as a modality for inducing changes in body composition, aerobic fitness, and muscular strength: a pilot study. Clin Interv Aging. 2014;9: 63–72. 10.2147/CIA.S30048 24399871 PMC3875193

[41] Lin C-I, Huang W-C, Chen W-C, Kan N-W, Wei L, Chiu Y-S, et al Effect of whole-body vibration training on body composition, exercise performance and biochemical responses in middle-aged mice. Metabolism. Elsevier Inc.; 2015;64: 1146–1156. 10.1016/j.metabol.2015.05.007 26045298

[42] Huang C, Tseng T, Huang W, Chung Y, Chuang H. Whole-Body Vibration Training Effect on Physical Performance and Obesity in Mice. 2014;11 10.7150/ijms.9975 PMC4196122 25317067

[43] Von Stengel S, Kemmler W, Engelke K, Kalender W a. Effects of whole body vibration on bone mineral density and falls: Results of the randomized controlled ELVIS study with postmenopausal women. Osteoporos Int. 2011;22: 317–325. 10.1007/s00198-010-1215-4 20306017

[44] Houtkooper RH, Argmann C, Houten SM, Cantó C, Jeninga EH, Andreux P a., et al The metabolic footprint of aging in mice. Sci Rep. 2011;1: 134 10.1038/srep00134 22355651 PMC3216615

[45] Rizzo MR, Mari D, Barbieri M, Ragno E, Grella R, Provenzano R, et al Resting metabolic rate and respiratory quotient in human longevity. J Clin Endocrinol Metab. 2005;90: 409–413. 10.1210/jc.2004-0390 15483081

[46] Zhao L, Zou X, Feng Z, Luo C, Liu J, Li H, et al Evidence for association of mitochondrial metabolism alteration with lipid accumulation in aging rats. Exp Gerontol. Elsevier Inc.; 2014;56: 3–12. 10.1016/j.exger.2014.02.001 24518876

[47] Badin PM, Langin D, Moro C. Dynamics of skeletal muscle lipid pools. Trends Endocrinol Metab. Elsevier Ltd; 2013;24: 607–615. 10.1016/j.tem.2013.08.001 23988586

[48] Barazzoni R, Short KR, Nair KS. Effects of aging on mitochondrial DNA copy number and cytochrome c oxidase gene expression in rat skeletal muscle, liver, and heart. J Biol Chem. 2000;275: 3343–3347. 10.1074/jbc.275.5.3343 10652323

[49] Kang C, Chung E, Diffee G, Ji LL. Exercise training attenuates aging-associated mitochondrial dysfunction in rat skeletal muscle: Role of PGC-1alpha. Exp Gerontol. 2013;48: 1343–1350. 10.1016/j.exger.2013.08.004 23994518

[50] Stuermer EK, Komrakova M, Werner C, Wicke M, Kolios L, Sehmisch S, et al Musculoskeletal response to whole-body vibration during fracture healing in intact and ovariectomized rats. Calcif Tissue Int. 2010;87: 168–180. 10.1007/s00223-010-9381-0 20532877 PMC2903688

[51] Andrews ZB, Horvath TL. Uncoupling protein-2 regulates lifespan in mice. Am J Physiol Endocrinol Metab. 2009;296: E621–E627. 10.1152/ajpendo.90903.2008 19141680 PMC2670629

[52] Barazzoni R, Nair KS. Changes in uncoupling protein-2 and -3 expression in aging rat skeletal muscle, liver, and heart. Am J Physiol Endocrinol Metab. 2001;280: E413–E419. 11171595 10.1152/ajpendo.2001.280.3.E413

[53] Vozza A, Parisi G, De Leonardis F, Lasorsa FM, Castegna A, Amorese D, et al UCP2 transports C4 metabolites out of mitochondria, regulating glucose and glutamine oxidation. Proc Natl Acad Sci U S A. 2014;111: 960–5. 10.1073/pnas.1317400111 24395786 PMC3903233

[54] Papa S, Skulachev VP. Reactive oxygen species, mitochondria, apoptosis and aging. Mol Cell Biochem. 1997;174: 305–19. 9309704

[55] Echtay KS, Roussel D, St-Pierre J, Jekabsons MB, Cadenas S, Stuart J a, et al Superoxide activates mitochondrial uncoupling proteins. Nature. 2002;415: 96–99. 10.1038/415096a 11780125

[56] Divakaruni AS, Brand MD. The regulation and physiology of mitochondrial proton leak. Physiology (Bethesda). 2011;26: 192–205. 10.1152/physiol.00046.2010 21670165

[57] Ando H, Noguchi R. Dependence of palmar sweating response and central nervous system activity on the frequency of whole-body vibration. Scand J Work Environ Heal. 2003;29: 216–219. 10.5271/sjweh.724 12828391

[58] Nagase I, Yoshida T, Saito M. Up-regulation of uncoupling proteins by beta-adrenergic stimulation in L6 myotubes. FEBS Lett. 2001;494: 175–80. 11311236 10.1016/s0014-5793(01)02341-9

[59] Lameloise N, Muzzin P, Prentki M, Assimacopoulos-Jeannet F. Uncoupling protein 2: a possible link between fatty acid excess and impaired glucose-induced insulin secretion? Diabetes. 2001;50: 803–809. 10.2337/diabetes.50.4.803 11289045

[60] Samec S, Seydoux J, Dulloo AG. Post-starvation gene expression of skeletal muscle uncoupling protein 2 and uncoupling protein 3 in response to dietary fat levels and fatty acid composition. 1999;17: 1339–1348. 10.2337/diabetes.48.2.436 10334328

[61] Winkler E, Klingenberg M. Effect of fatty acids on H+ transport activity of the reconstituted uncoupling protein. J Biol Chem. 1994;269: 2508–2515. 8300577

