# Supplementary figures and images for: Whole-Body Vibration Partially Reverses Aging-Induced Increases in Visceral Adiposity and Hepatic Lipid Storage in Mice
Source: PLoS One. 2016 Feb 17;11(2):e0149419. doi: 10.1371/journal.pone.0149419 (PMC4757540; doi:10.1371/journal.pone.0149419)

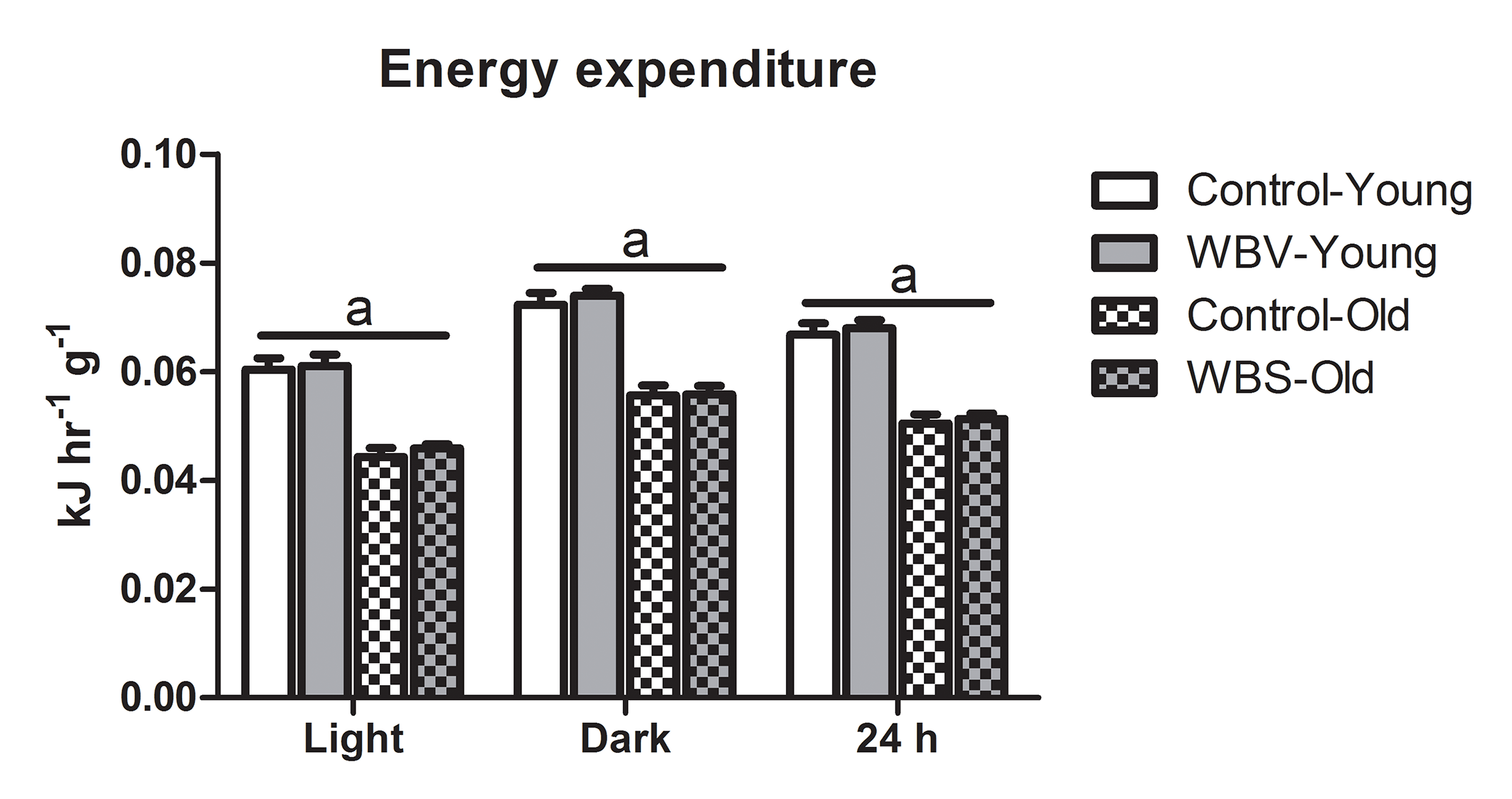

Supplement: S1 Fig — Data are averages from n = 7–8 mice per group; ± SEM. ap<0.05, significant effect of age. (TIF) [file pone.0149419.s001.tif]

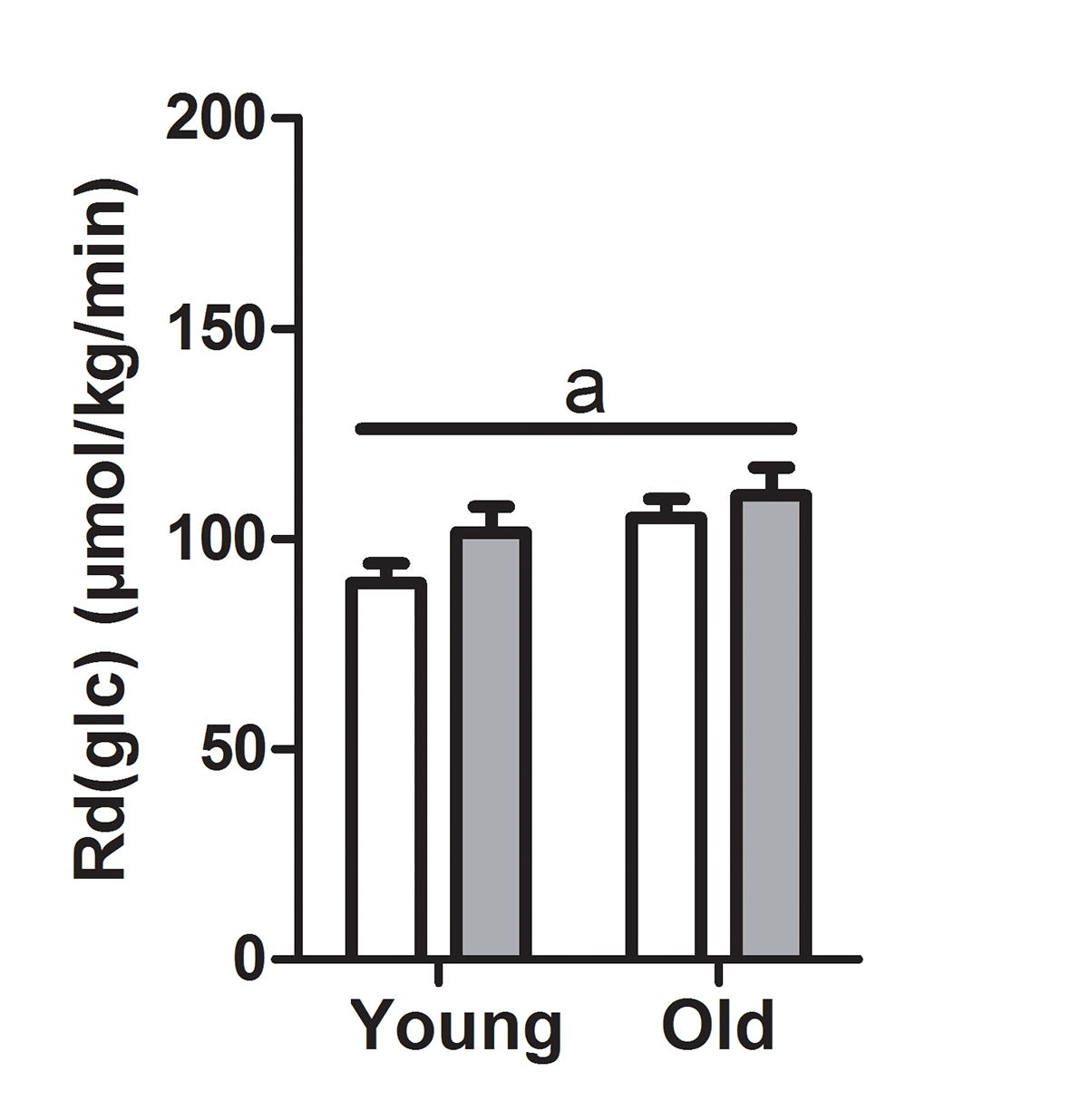

Supplement: S2 Fig — Data are averages from n = 6–9 mice per group; ± SEM. ap<0.05, significant effect of age. (TIF) [file pone.0149419.s002.tif]
